# Meta-Analysis of 125 Rheumatoid Arthritis-Related Single Nucleotide Polymorphisms Studied in the Past Two Decades

**DOI:** 10.1371/journal.pone.0051571

**Published:** 2012-12-10

**Authors:** Yongshuai Jiang, Ruijie Zhang, Jiajia Zheng, Panpan Liu, Guoping Tang, Hongchao Lv, Lanying Zhang, Zhenwei Shang, Yuanbo Zhan, Wenhua Lv, Miao Shi, Ruimin Zhang

**Affiliations:** 1 College of Bioinformatics Science and Technology, Harbin Medical University, Harbin, China; 2 The Second Affiliated Hospital of Harbin Medical University, Harbin, China; Keio University School of Medicine, Japan

## Abstract

**Objective:**

Candidate gene association studies and genome-wide association studies (GWAs) have identified a large number of single nucleotide polymorphisms (SNPs) loci affecting susceptibility to rheumatoid arthritis (RA). However, for the same locus, some studies have yielded inconsistent results. To assess all the available evidence for association, we performed a meta-analysis on previously published case-control studies investigating the association between SNPs and RA.

**Methods:**

Two hundred and sixteen studies, involving 125 SNPs, were reviewed. For each SNP, three genetic models were considered: the allele, dominant and recessive effects models. For each model, the effect summary odds ratio (*OR*) and 95% CIs were calculated. Cochran’s *Q*-statistics were used to assess heterogeneity. If the heterogeneity was high, a random effects model was used for meta-analysis, otherwise a fixed effects model was used.

**Results:**

The meta-analysis results showed that: (1) 30, 28 and 26 SNPs were significantly associated with RA (*P*<0.01) for the allele, dominant, and recessive models, respectively. (2) rs2476601 (PTPN22) showed the strongest association for all the three models: *OR* = 1.605, 95% CI: 1.540–1.672, P<1.00E−15 for the T-allele; *OR* = 1.638, 95% CI: 1.565–1.714, P<1.00E−15 for the T/T+T/C genotype and *OR* = 2.544, 95% CI: 2.173–2.978, *P<*1.00E−15 for the T/T genotype. (3) Only 23 (18.4%), 13 (10.4%) and 15 (12.0%) SNPs had high heterogeneity (*P*<0.01) for the three models, respectively. (4) For some of the SNPs, there was no publication bias according to Funnel plots and Egger’s regression tests (P<0.01). For the other SNPs, the associations were tested in only a few studies, and may have been subject to publication bias. More studies on these loci are required.

**Conclusion:**

Our meta-analysis provides a comprehensive evaluation of the RA association studies from the past two decades. The detailed meta-analysis results are available at: http://210.46.85.180/DRAP/index.php/Metaanalysis/index.

## Introduction

Rheumatoid Arthritis (RA) is an autoimmune disease that causes inflammation of the joints and surrounding tissues. Its main symptoms are pain, swelling, stiffness and loss of function in the joints [1]. The prevalence of RA is about 1% in the adult population, and is higher among women than men [2].

As a common, complex disease, RA is usually caused by the interaction of multiple genetic variants and environmental factors [3]. Based on twin studies, the contribution of genetic factors is estimated to account for about 50–65% of the risk of developing RA [4], [5]. Therefore, the identification of genetic factors is important for understanding the pathogenesis of RA.

Many studies have successfully identified RA disease loci. The most significant genetic locus for RA is the human leukocyte antigen (HLA) within the major histocompatibility complex (MHC) on chr 6p21. There are many alleles of the HLA class II gene, *DRB1*, especially affecting a shared common string of amino acid residues (the shared-epitope, SE). These DRB1 alleles have consistently been shown to have strong association with RA [6], [7]. However, the region has a highly complex genetic structure, which hinders the effectiveness of standard SNP-based genotyping and analysis [8]. Family studies also suggest that the HLA region only contributes one-third of the genetic component [9]. Therefore, non-HLA loci associated with RA are being increasingly studied. SNP-based association studies (including candidate gene association studies and genome-wide association studies-GWAs) are effective for identifying those non-HLA loci. The number of association studies has grown rapidly year on year, and many important genes, such as PTPN22 and STAT4, have been successfully identified [10].

Although association studies of RA have achieved great success, certain problems remain. For the same locus, some studies have yielded conflicting results. For example, Munoz-Valle et al. described that the SNP rs231775 at position 49 (A/G) of the CTLA-4 gene is associated with RA [11]. However, Milicic et al., indicated that rs231775 is not associated with susceptibility to RA [12]. The inconsistent results may be caused by small sample sizes, racial or ethnic differences, and clinical or genetic heterogeneity [13]. Therefore, it is important to assess whether the combined evidence would show associations between SNPs and RA.

Meta-analysis is a powerful tool that can improve the statistical performance by combining the results of multiple studies. Using meta-analysis methods, certain SNP loci, such as STAT4 rs7574865 [14], [15], PADI4 rs2240340 [16], and PTPN22 rs2476601 [17], [18], have been evaluated for their association with RA. However, each meta-analysis report only involved one or a few SNP loci. To comprehensively and systematically assess the associations between all available SNPs (each SNP was reported by multiple RA case-control association studies) and RA susceptibility, we searched the PubMed database, and performed a meta-analysis. One hundred and twenty five SNPs were included in our study. For each SNP, three genetic models were considered: the allele model, dominant model and recessive model. Heterogeneity and publication bias were also assessed. As far as we know, this is the most detailed meta-analysis report of RA-related SNPs yet published.

**Table 1:** Meta-analysis results under the allele model.

SNP	Comparison (A vs. a)	Gene symbol	No. of studies	*Q*	*Q*_*P*	*I^2^*	aModel	b *OR* (95%CI)	Meta_*P*
rs2476601	T vs. C	PTPN22	34	44.153	0.093	0.253	fixed	1.605 (1.540, 1.672)	<1.00E−15
rs7574865	T vs. G	STAT4	12	18.125	0.079	0.393	fixed	1.287 (1.225, 1.353)	<1.00E−15
rs2488457	C vs. G	PTPN22	3	6.612	0.037	0.698	fixed	1.467 (1.278, 1.685)	5.31E−08
rs6920220	A vs. G	TNFAIP3	4	1.746	0.627	0.000	fixed	1.211 (1.128, 1.300)	1.38E−07
rs4112788	C vs. T	LCE3C	2	0.156	0.693	0.000	fixed	1.247 (1.133, 1.371)	6.03E−06
rs33996649	G vs. A	PTPN22	8	2.659	0.915	0.000	fixed	1.258 (1.136, 1.392)	9.26E−06
rs1217413	C vs. T	PTPN22	2	0.212	0.645	0.000	fixed	1.383 (1.187, 1.610)	3.03E−05
rs11889341	T vs. C	STAT4	3	2.267	0.322	0.118	fixed	1.214 (1.108, 1.331)	3.07E−05
rs874881	G vs. C	PADI4	7	3.086	0.798	0.000	fixed	1.204 (1.101, 1.316)	4.67E−05
rs7021206	A vs. G	TRAF1	2	0.192	0.661	0.000	fixed	0.810 (0.731, 0.898)	6.25E−05
rs10181656	G vs. C	STAT4	2	1.737	0.188	0.424	fixed	1.255 (1.120, 1.405)	8.71E−05
rs8179673	C vs. T	STAT4	2	0.960	0.327	0.000	fixed	1.253 (1.119, 1.402)	9.01E−05
rs231775	G vs. A	CTLA4	12	14.692	0.197	0.251	fixed	1.142 (1.068, 1.220)	9.33E−05
rs396991	V vs. F	FCGR3A	10	13.201	0.154	0.318	fixed	1.192 (1.090, 1.303)	1.22E−04
rs6498169	G vs. A	CLEC16A	2	0.422	0.516	0.000	fixed	1.208 (1.095, 1.334)	1.70E−04
rs6822844	G vs. T	IL2	4	6.631	0.085	0.548	fixed	1.221 (1.094, 1.362)	3.70E−04
PADI4_97	2 vs. 1	PADI4	2	0.512	0.474	0.000	fixed	1.290 (1.118, 1.487)	4.72E−04
rs3087243	G vs. A	CTLA4	3	5.767	0.056	0.653	fixed	1.161 (1.065, 1.266)	7.00E−04
rs2488458	A vs. G	PTPN22	2	0.242	0.623	0.000	fixed	1.265 (1.094, 1.463)	1.49E−03
rs11203366	G vs. A	PADI4	4	9.539	0.023	0.686	fixed	1.224 (1.077, 1.391)	1.91E−03
rs13207033	2 vs. 1	TNFAIP3	2	6.205	0.013	0.839	fixed	0.895 (0.835, 0.961)	2.06E−03
PADI4_99	2 vs. 1	PADI4	2	6.198	0.013	0.839	fixed	1.238 (1.079, 1.421)	2.37E−03
MIF -173G/C	C vs. G	AMH	2	0.038	0.845	0.000	fixed	1.295 (1.094, 1.532)	2.64E−03
rs7528684	C vs. T	FCRL3	15	30.192	0.007	0.536	random	1.093 (1.031, 1.158)	2.95E−03
TAP2 565A/G	G vs. A	TAP2	2	0.680	0.410	0.000	fixed	0.519 (0.334, 0.805)	3.45E−03
rs3811021	T vs. C	PTPN22	3	0.926	0.630	0.000	fixed	1.199 (1.060, 1.356)	3.79E−03
rs1748033	T vs. C	PADI4	9	24.614	0.002	0.675	random	1.223 (1.066, 1.404)	4.10E−03
rs360722	C vs. T	IL18	2	0.000	0.995	0.000	fixed	1.176 (1.052, 1.316)	4.49E−03
rs11203367	T vs. C	PADI4	4	7.043	0.071	0.574	fixed	1.202 (1.059, 1.365)	4.57E−03
rs231779	T vs. C	CTLA4	2	0.966	0.326	0.000	fixed	1.151 (1.037, 1.279)	8.38E−03

a Model: If the *Q-*statistic was significant (*P*<0.01), we selected random effects model for a meta-analysis, otherwise we selected fixed effects model.

b *OR* = Combined odds ratio.

## Materials and Methods

### Data Collection

The PubMed literature database was used to search for appropriate studies. The following key words were used: ‘polymorphism’, ‘single nucleotide polymorphisms’, ‘genome-wide association study’, ‘GWAs’, ‘rheumatoid arthritis’ and ‘RA’. All the studies were selected in accordance with following criteria: (1) all the articles were published between January 1992 and December 2011; (2) all the studies must be a case-control design, and examine the association between SNPs and RA; (3) the data of SNP genotypes in patients and in controls was available; and (4) the study was published as a full paper, not as an meeting abstract or review. Ultimately, 216 studies involving 125 SNPs were included in the meta-analysis. For each study, the following information was extracted: the polymorphism studied, the first author, year of publication, demographics, the numbers of cases and controls for the study.

**Table 2:** Meta-analysis results under the dominant model.

SNP	Comparison(AA+Aa vs. aa)	Gene symbol	No. of studies	*Q*	*Q_P*	*I^2^*	aModel	b *OR* (95%CI)	Meta_*P*
rs2476601	TT+TC vs. CC	PTPN22	34	45.474	0.073	0.274	fixed	1.638 (1.565, 1.714)	<1.00E−15
rs7574865	TT+TG vs. GG	STAT4	12	9.143	0.609	0.000	fixed	1.355 (1.269, 1.447)	<1.00E−15
rs2488457	CC+CG vs. GG	PTPN22	3	4.863	0.088	0.589	fixed	1.512 (1.274, 1.795)	2.30E−06
rs6920220	AA+AG vs. GG	TNFAIP3	4	1.751	0.626	0.000	fixed	1.228 (1.128, 1.337)	2.34E−06
rs874881	GG+GC vs. CC	PADI4	7	5.483	0.484	0.000	fixed	1.329 (1.166, 1.514)	1.93E−05
rs1217413	CC+CT vs. TT	PTPN22	2	0.000	0.995	0.000	fixed	1.460 (1.213, 1.757)	6.34E−05
rs2227309	AA+AG vs. GG	CASP7	5	4.948	0.293	0.192	fixed	0.742 (0.637, 0.866)	1.41E−04
rs360722	CC+CT vs. TT	IL18	2	3.004	0.083	0.667	fixed	1.433 (1.184, 1.734)	2.23E−04
rs11889341	TT+TC vs. CC	STAT4	3	0.203	0.903	0.000	fixed	1.260 (1.113, 1.426)	2.50E−04
rs231775	GG+GA vs. AA	CTLA4	12	14.210	0.222	0.226	fixed	1.212 (1.093, 1.344)	2.55E−04
rs8179673	CC+CT vs. TT	STAT4	2	1.780	0.182	0.438	fixed	1.299 (1.110, 1.519)	1.11E−03
rs2812378	CC+CT vs. TT	CCL21	3	0.263	0.877	0.000	fixed	1.151 (1.055, 1.256)	1.55E−03
rs10181656	GG+GC vs. CC	STAT4	2	1.711	0.191	0.416	fixed	1.287 (1.101, 1.505)	1.56E−03
rs396991	VV+VF vs. FF	FCGR3A	10	11.620	0.236	0.225	fixed	1.212 (1.074, 1.368)	1.83E−03
rs7528684	CC+CT vs. TT	FCRL3	15	21.020	0.101	0.334	fixed	1.096 (1.034, 1.162)	2.01E−03
rs231779	TT+TC vs. CC	CTLA4	2	0.001	0.970	0.000	fixed	1.283 (1.095, 1.503)	2.05E−03
rs1805010	GG+GA vs. AA	IL4R	2	2.786	0.095	0.641	fixed	1.330 (1.107, 1.597)	2.27E−03
rs767455	AA+AG vs. GG	TNFRSF1A	3	3.936	0.140	0.492	fixed	1.664 (1.194, 2.319)	2.62E−03
rs6498169	GG+GA vs. AA	CLEC16A	2	0.140	0.708	0.000	fixed	1.236 (1.076, 1.419)	2.77E−03
rs11203367	TT+TC vs. CC	PADI4	4	9.562	0.023	0.686	fixed	1.316 (1.095, 1.582)	3.37E−03
rs2488458	AA+AG vs. GG	PTPN22	2	1.361	0.243	0.265	fixed	1.310 (1.092, 1.571)	3.68E−03
PDCD1 PD-1.1 G/A	GG+GA vs. AA	PDCD1	2	4.332	0.037	0.769	fixed	1.357 (1.102, 1.671)	4.02E−03
MIF -173G/C	CC+CG vs. GG	AMH	2	0.123	0.725	0.000	fixed	1.309 (1.083, 1.583)	5.35E−03
rs10818488	GG+GA vs. AA	TRAF1	4	5.427	0.143	0.447	fixed	0.862 (0.774, 0.959)	6.48E−03
rs13207033	22+21 vs. 11	TNFAIP3	2	6.429	0.011	0.844	fixed	0.887 (0.814, 0.967)	6.60E−03
TAP2 565A/G	GG+GA vs. AA	TAP2	2	0.170	0.680	0.000	fixed	0.063 (0.008, 0.475)	7.27E−03
rs1748033	TT+TC vs. CC	PADI4	9	20.260	0.009	0.605	random	1.263 (1.063, 1.500)	7.83E−03
rs11203366	GG+GA vs. AA	PADI4	4	6.454	0.091	0.535	fixed	1.283 (1.065, 1.544)	8.56E−03

a Model: If the *Q-*statistic was significant (P<0.01), we selected random effects model for a meta-analysis, otherwise we selected fixed effects model.

b *OR* = Combined odds ratio.

### Selection of the Genetic Model

To comprehensively analyze the relationships between SNPs and RA, three genetic models were selected: the allele model, the dominant model, and the recessive model. To illustrate the models, we assumed that a SNP marker locus has two alleles, labeled *A* and *a* (SNPs normally have only two alleles). *A* is the high-risk candidate allele and *a* is the lower-risk allele. Three models are described as follows:

1. Allele model: The effect of the *A* allele vs. the *a* allele.
2. Dominant model: If it produces an RA phenotype when present in either one or two copies of *A* allele, that is, the *A/A+A/a vs*. the *a/a* genotypes
3. Recessive model: If it produces a RA phenotype only when present in two copies of *A* allele, that is, the *A/A vs.* the *A/a+a/a* genotypes

For each model, we calculated the *OR* and its 95% CI for the individual study.

**Table 3:** Meta-analysis results under the recessive model.

SNP	Comparison (AA vs. Aa+aa)	Gene symbol	No. of studies	*Q*	*Q*_*P*	*I^2^*	aModel	b *OR* (95%CI)	Meta_*P*
rs2476601	TT vs. CC+TC	PTPN22	34	25.180	0.833	0.000	fixed	2.544 (2.173, 2.978)	<1.00E−15
rs7574865	TT vs. GG+TG	STAT4	12	22.683	0.020	0.515	fixed	1.419 (1.278, 1.577)	6.71E−11
rs4112788	CC vs. TT+CT	LCE3C	2	0.300	0.584	0.000	fixed	1.445 (1.255, 1.664)	2.95E−07
rs33996649	GG vs. AA+GA	PTPN22	8	2.373	0.936	0.000	fixed	1.289 (1.163, 1.430)	1.40E−06
rs2240340	TT vs. CC+TC	PADI4	7	16.385	0.012	0.634	fixed	1.198 (1.100, 1.304)	3.37E−05
rs7021206	AA vs. GG+AG	TRAF1	2	0.497	0.481	0.000	fixed	0.763 (0.668, 0.871)	6.27E−05
rs2488457	CC vs. GG+CG	PTPN22	3	7.256	0.027	0.724	fixed	2.019 (1.422, 2.867)	8.55E−05
rs1748033	TT vs. CC+TC	PADI4	9	13.040	0.110	0.386	fixed	1.326 (1.151, 1.528)	9.57E−05
rs6822844	GG vs. TT+GT	IL2	4	4.718	0.194	0.364	fixed	1.278 (1.127, 1.448)	1.24E−04
rs6920220	AA vs. GG+AG	TNFAIP3	4	0.218	0.975	0.000	fixed	1.466 (1.198, 1.796)	2.12E−04
rs2073838	AA vs. GG+AG	SLC22A4	4	10.681	0.014	0.719	fixed	1.361 (1.147, 1.616)	4.22E−04
rs2243250	TT vs. CC+TC	IL4	6	2.184	0.823	0.000	fixed	2.254 (1.433, 3.546)	4.38E−04
PADI4_97	22 vs. 11+21	PADI4	2	0.107	0.743	0.000	fixed	1.706 (1.261, 2.307)	5.25E−04
rs10181656	GG vs. CC+GC	STAT4	2	1.010	0.315	0.010	fixed	1.486 (1.176, 1.878)	9.18E−04
rs396991	VV vs. FF+VF	FCGR3A	10	8.443	0.490	0.000	fixed	1.372 (1.133, 1.661)	1.18E−03
rs6498169	GG vs. AA+GA	CLEC16A	2	0.261	0.610	0.000	fixed	1.375 (1.132, 1.670)	1.32E−03
rs8179673	CC vs. TT+CT	STAT4	2	0.005	0.942	0.000	fixed	1.435 (1.144, 1.799)	1.79E−03
rs11889341	TT vs. CC+TC	STAT4	3	5.248	0.073	0.619	fixed	1.341 (1.109, 1.622)	2.50E−03
rs3087243	GG vs. AA+GA	CTLA4	3	7.377	0.025	0.729	fixed	1.203 (1.066, 1.358)	2.72E−03
rs3811021	TT vs. CC+TC	PTPN22	3	0.907	0.635	0.000	fixed	1.227 (1.062, 1.417)	5.45E−03
IL-2 -330T/G	GG vs. TT+GT	IL2	2	1.970	0.160	0.492	fixed	2.062 (1.233, 3.447)	5.79E−03
rs1801133	TT vs. CC+TC	MTHFR	5	5.504	0.239	0.273	fixed	0.721 (0.565, 0.920)	8.66E−03
rs7528684	CC vs. TT+CT	FCRL3	15	36.084	0.001	0.612	random	1.168 (1.040, 1.311)	8.77E−03
PADI4_99	22 vs. 11+21	PADI4	2	5.313	0.021	0.812	fixed	1.401 (1.088, 1.803)	8.89E−03
rs231775	GG vs. AA+GA	CTLA4	12	14.891	0.188	0.261	fixed	1.158 (1.036, 1.295)	9.63E−03
rs3789604	AA vs. CC+AC	PTPN22	3	0.707	0.702	0.000	fixed	1.243 (1.054, 1.468)	9.98E−03

a Model: If the *Q-*statistic was significant (P<0.01), we selected random effects model for a meta-analysis, otherwise we selected fixed effects model.

b *OR* = Combined odds ratio.

### Evaluation of the Heterogeneity

Cochran’s *Q*-statistics were used to test the heterogeneity of between- and within-study variation [19]. The statistics follow a 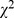 distribution with k-1 degrees of freedom (where k is the number of studies). The null hypothesis was that all studies were evaluating the same effect. Rejecting the null hypothesis means heterogeneity exists between studies. The significance level was 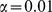.

Another indicator, *I^2^*, was used to measure the degree of inconsistency across studies. *I^2^* is given by the formula: 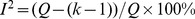 (where k is the number of studies). It measures the percentage of total variation across studies caused by heterogeneity rather than by chance [20]. *I^2^* takes values between 0% and 100%. *I^2^ = *0–25% indicates low heterogeneity; *I^2^ = *25–50% indicates moderate heterogeneity, *I^2^ = *50–75% indicates large heterogeneity and *I^2^ = *75–100% indicates extreme heterogeneity [21], [22].

**Table 4:** Meta-analysis results of special phenotypes.

SNP	Gene symbol	Type of study	Comparison	No. of studies	Genetic model	Heterogeneity Test (*P*)	aModel	b *OR* (95%CI)	Meta_*P*
rs2476601	PTPN22	RF+/control	T vs. C	10	allele	0.873	fixed	1.672 (1.513, 1.848)	<1.00E−15
rs2476601	PTPN22	CPP+/control	T vs. C	3	allele	0.064	fixed	1.981 (1.582, 2.479)	2.41E−09
rs2476601	PTPN22	RF−/control	T vs. C	9	allele	0.030	fixed	1.377 (1.194, 1.587)	1.05E−05
rs2476601	PTPN22	RF+/RF-	T vs. C	2	allele	0.724	fixed	1.437 (1.161, 1.778)	8.48E−04
rs2476601	PTPN22	RF+/control	TT+TC vs. CC	10	dominant	0.833	fixed	1.692 (1.515, 1.890)	<1.00E−15
rs2476601	PTPN22	CPP+/control	TT+TC vs. CC	3	dominant	0.043	fixed	2.070 (1.609, 2.664)	1.52E−08
rs2476601	PTPN22	RF−/control	TT+TC vs. CC	9	dominant	0.019	fixed	1.400 (1.197, 1.637)	2.49E−05
rs2476601	PTPN22	RF+/RF-	TT+TC vs. CC	2	dominant	0.838	fixed	1.426 (1.128, 1.803)	3.03E−03
rs2476601	PTPN22	RF+/control	TT vs. CC+TC	10	recessive	0.492	fixed	2.784 (1.945, 3.985)	2.16E−08
rs2476601	PTPN22	CPP+/control	TT vs. CC+TC	3	recessive	0.809	fixed	3.519 (1.548, 8.000)	2.67E−03
rs7021206	TRAF1	RF+/control	G vs. A	2	allele	0.182	fixed	1.274 (1.146, 1.416)	7.16E−06
rs7021206	TRAF1	CPP+/control	G vs. A	2	allele	0.203	fixed	1.292 (1.151, 1.450)	1.43E−05
rs7021206	TRAF1	CPP−/control	G vs. A	2	allele	0.645	fixed	1.289 (1.082, 1.535)	4.38E−03
rs7021206	TRAF1	RF+/control	GG+GA vs. AA	2	dominant	0.134	fixed	1.346 (1.174, 1.543)	2.00E−05
rs7021206	TRAF1	CPP−/control	GG+GA vs. AA	2	dominant	0.952	fixed	1.372 (1.089, 1.730)	7.40E−03
rs7021206	TRAF1	CPP+/control	GG+GA vs. AA	2	dominant	0.204	fixed	1.394 (1.200, 1.621)	1.49E−05
rs7021206	TRAF1	RF+/control	GG vs. AA+AG	2	recessive	0.575	fixed	1.429 (1.112, 1.836)	5.30E−03
rs7574865	STAT4	RF+/control	T vs. G	4	allele	0.492	fixed	1.466 (1.298, 1.655)	6.65E−10
rs7574865	STAT4	RF−/control	T vs. G	4	allele	0.858	fixed	1.465 (1.259, 1.706)	7.96E−07
rs7574865	STAT4	CPP+/control	T vs. G	3	allele	0.586	fixed	1.474 (1.247, 1.741)	5.24E−06
rs7574865	STAT4	CPP−/control	T vs. G	3	allele	0.239	fixed	1.446 (1.192, 1.755)	1.84E−04
rs7574865	STAT4	RF+/control	TT+TG vs. GG	4	dominant	0.482	fixed	1.516 (1.303, 1.764)	6.95E−08
rs7574865	STAT4	RF−/control	TT+TG vs. GG	4	dominant	0.861	fixed	1.522 (1.258, 1.841)	1.56E−05
rs7574865	STAT4	CPP+/control	TT+TG vs. GG	3	dominant	0.743	fixed	1.517 (1.227, 1.874)	1.13E−04
rs7574865	STAT4	CPP−/control	TT+TG vs. GG	3	dominant	0.551	fixed	1.386 (1.082, 1.775)	9.78E−03
rs7574865	STAT4	RF+/control	TT vs. GG+TG	4	recessive	0.385	fixed	2.019 (1.490, 2.738)	5.99E−06
rs7574865	STAT4	CPP+/control	TT vs. GG+TG	3	recessive	0.082	fixed	2.463 (1.604, 3.782)	3.82E−05
rs7574865	STAT4	CPP−/control	TT vs. GG+TG	3	recessive	0.212	fixed	2.120 (1.417, 3.172)	2.54E−04
rs7574865	STAT4	RF−/control	TT vs. GG+TG	4	recessive	0.146	fixed	1.987 (1.370, 2.882)	2.94E−04

RF+: RF positive; RF-: RF negative; CPP+: anti-CCP positive; CPP-: anti-CCP negative.

a Model: If the *Q-*statistic was significant (P<0.01), we selected random effects model for a meta-analysis, otherwise we selected fixed effects model.

b *OR* = Combined odds ratio.

### Evaluation of the Statistical Association

For each of the 125 loci, Cochran’s *Q*-statistics were used to test heterogeneity. If the *Q*-statistic was not significant, all differences between studies were considered to be caused by sampling error. Then, a fixed effects model was selected for the meta-analysis. The fixed effects model assumes that all studies in the meta-analysis share a common effect size. In contrast, if the *Q*-statistic was significant (*P<*0.01), heterogeneity existed between the studies. In this case, a random effects model was selected for the meta-analysis. The random effects model assumes that each study has a specific effect size, and allows heterogeneity exists between studies [23].

### Evaluation of Publication Bias

Funnel plots were used to assess publication bias. The estimated effects were plotted against their standard error. Usually, larger sample studies have smaller standard errors, and smaller sample studies have larger standard errors. Therefore, the estimated effects of small studies are more widely scattered than those of larger studies. If there is no bias, the plot will be a symmetrical inverted funnel. Egger’s test was used to test the asymmetry of the funnel plot [24], [25].

**Table 5:** Meta-analysis results of subgroups.

SNP	Gene symbol	Risk allele	Subgroup	Comparison	No. of studies	Heterogeneity Test (*P*)	aModel	b *OR* (95%CI)	Meta_*P*
rs7528684	FCRL3	C	Total	C vs. T	15	0.007	random	1.09 (1.03, 1.16)	2.95E−03
		C	European	C vs. T	5	0.204	fixed	1.05 (0.99, 1.12)	0.100
		C	Asian	C vs. T	6	0.049	fixed	1.17 (1.09, 1.24)	<1.00E−4
		C	American	C vs. T	4	0.132	fixed	1.02 (0.94, 1.12)	0.572
rs1800629	TNF	A	Total	A vs. G	14	<1.00E−4	random	0.99 (0.88, 1.13 )	0.931
		A	European	A vs. G	6	0.148	fixed	0.97 (0.83, 1.14)	0.721
		G	Asian	G vs. A	4	0.011	fixed	2.17 (1.61, 2.92 )	<1.00E−4
		A	American	A vs. G	3	0.574	fixed	1.91 (1.40, 2.62)	<1.00E−4
rs1800896	IL10	G	Total	G vs. A	11	0.004	random	0.99 (0.84, 1.17)	0.928
		G	European	G vs. A	8	0.001	random	0.97 (0.78, 1.22)	0.824

a Model: If the *Q-*statistic was significant (P<0.01), we selected random effects model for a meta-analysis, otherwise we selected fixed effects model.

b *OR* = Combined odds ratio.

All statistical analyzes were performed using ‘Meta’ packages in the R language (http://cran.r-project.org/web/packages/meta/index.html).

## Results

### Eligible Studies and Loci

The PubMed database was searched and about 1,500 studies were reviewed. Ultimately, 216 published articles involving 125 SNPs were included in the meta-analysis. Each SNP was reported in at least two studies. The number of studies for each locus was also counted. Twenty SNPs were reported more than five times, 41 SNPs were reported three to four times, and 64 SNPs were reported twice. SNP rs2476601 at the *PTPN22* gene locus was reported most frequently (34 times). For each study, the number of SNP genotypes in cases and controls was extracted for subsequent analysis.

### Meta-analysis Results under the Allele Genetic Model

For each SNP, the *OR* and its 95% CI of the A allele (A vs. a) were calculated for the individual study, and the heterogeneity between studies was tested.

After heterogeneity testing, 23 SNPs had a *Q*-statistic *P*<0.01. For the meta-analysis of these SNPs, a random effects model was used. For the remaining 102 SNPs, a fixed effects model was used. The meta-analysis showed that 30 SNPs were significantly associated with RA (*P*<0.01, see Table 1). Among these 30 SNPs, only two showed heterogeneity (p = 0.007 and 0.002 for rs7528684 and rs1748033). For these two loci, the overall *OR* based on the random effects model were 1.093 (95% CI: 1.031–1.158) for rs7528684 and 1.223 (95% CI 1.066–1.404) for rs1748033. The other 28 SNPs showed an association with RA under the fixed effects model. The most significant locus is rs2476601 (*PTPN22* risk allele 1858T). The pooled summary *OR* based on the fixed effects model was 1.605 (95% CI: 1.540–1.672), suggesting that the rs2476601 T-allele does confer susceptibility to RA. The publication bias was tested using Egger’s test. No significant publication biases were observed for any of the 30 significant SNPs.

### Meta-analysis Results under the Dominant Genetic Model

Based on the dominant model (AA+Aa vs. aa genotype), the heterogeneity between the studies was tested. Thirteen SNPs had high heterogeneity (*P*<0.01), and were analyzed using the random effects model. One hundred and twelve SNPs were analyzed using the fixed effects model. Table 2 lists all the 28 significantly associated SNPs. Among these SNPs, only rs1748033 showed heterogeneity (*Q* = 20.260, *P* = 0.009). For SNP rs7528684, although heterogeneity was observed under the allele model, it did not show heterogeneity under the dominant model (*Q* = 21.020, *P* = 0.101). Therefore, the random effects model was used for the meta-analysis of rs1748033, and fixed effects model for rs7528684. All the meta-analysis results were compared under the allele model and the dominant model. Twenty-two SNPs displayed associations with RA under both models. Six SNP (rs2227309, rs2812378, rs1805010, rs767455, PDCD1 PD-1.1 G/A and rs10818488) were associated with RA only under the dominant model rather than the allele model. SNP rs2476601 (*PTPN22* risk allele 1858T) still had the strongest association with RA under the dominant model (*OR* = 1.638; 95% CI = 1.565–1.714; P-value P<1.00E−15). For all 28 significant SNPs, no significant publication biases were observed.

### Meta-analysis Results using the Recessive Genetic Model

For the recessive model (AA vs. Aa+aa), 15 SNPs were analyzed using the random effects model, and 110 SNPs were analyzed using the fixed effects model. Ultimately, 26 SNPs displayed significant associations with RA (*P<*0.01, see Table 3). Among these 26 SNPs, 25 were assessed for an association with RA using the fixed effects model, and only one SNP, rs7528684, was assessed using the random effects model because of heterogeneity (*Q = *36.084, *P = *0.001). Compared with the allele model, 20 SNPs were significantly associated with RA under both the recessive model and the allele model, and six SNPs were specificity associated with RA under the recessive model. Compared with the dominant model, 12 SNPs were significantly associated with RA under both the recessive model and the dominant model, and 14 SNPs were specificity associated with RA under recessive model. Twelve SNPs (rs2476601, rs7574865, rs2488457, rs1748033, rs6920220, rs10181656, rs396991, rs6498169, rs8179673, rs11889341, rs7528684 and rs231775) were significantly associated with RA under all the three genetic models. The most significant locus was still rs2476601 (*PTPN22* risk allele 1858T; *OR* = 2.544; 95% CI = 2.173–2.978; P-value P<1.00E−15). For all 26 significant SNPs, no significant publication biases were observed.

More detailed meta-analysis results were gathered for each of the 125 loci, including: detailed list of articles (the first author, year of publication, demographics, the numbers of cases and controls for the study), individual and combed *OR* and 95% CI, results of Cochran *Q* test (*Q* and *P* values), *I^2^* and its 95% CI, results of meta-analysis (under the fixed effects model and the random effects model), forest plots and funnel plot for publication biases. These results are all available at: http://210.46.85.180/DRAP/index.php/Metaanalysis/index.

### Meta-analysis of Special Phenotypes

In the process of data collection, we noticed that some articles also provided additional testing for samples, such as rheumatoid factor (RF, positive or negative) and anti-cyclic citrullinated peptide antibody (anti-CCP, positive or negative). A meta-analysis of the SNPs that included the above information (16 SNPs) was also carried out. The meta-analysis results are shown in Table 4. Three (SNPs rs2476601, rs7021206 and rs7574865) were significantly associated with these phenotypes. For rs2476601 (*PTPN22* gene), the T allele was significantly associated with RA in RF-positive, RF-negative and anti-CCP-positive RA patients versus controls. In addition, the T allele also showed a significant difference between RF-positive and -negative subjects. For rs7021206 (*TRAF1* gene), the G allele was significantly associated with RA in RF-positive, anti-CCP-positive and anti-CCP-negative RA patients versus controls. For rs7574865 (*STAT4* gene), the T allele was significantly associated with RA in RF-positive, RF-negative, anti-CCP-positive, and anti-CCP- negative RA patients versus controls. No heterogeneity was found for any of the 16 SNPs, and the meta-analysis was performed using fixed effects model. For more detailed results for each SNP see: http://210.46.85.180/DRAP/index.php/Metaanalysis/index.

### Meta-analysis of Population Subgroups

In this paragraph, some of the SNPs that showed heterogeneity were subjected to subgroup analysis to explain the causes of the heterogeneity. Some SNPs were reported by a few individual studies, and not suitable for the classification by subgroups. Thus, SNPs were selected that were reported by more than 10 individual studies for the subgroup analysis. Three SNPs, rs7528684 (15 studies), rs1800629 (14 studies) and rs1800896 (11 studies) were selected. Only the allele model was considered. The meta-analysis results of the subgroups are shown in Table 5. For SNP rs7528684, 15 studies were divided into three subgroups: European (five studies), Asian (six studies) and American (four studies). For each subgroup, no heterogeneity was observed. The meta-analysis results showed that rs7528684 was associated with RA only in the Asian subgroup (*OR* = 1.17, 95% CI: 1.09–1.24, *P*<1.00E−4). No evidence of association was observed in European and American subgroups. This indicated that the heterogeneity of rs7528684 may be caused by regional differences, and the C allele is a risk allele in the Asian population, but not in Europeans and Americans. For SNP rs1800629, 14 studies were divided into three subgroups: European (six studies), Asian (four studies) and American (three studies). One study was excluded because the study population was African. For each of the three subgroups, no heterogeneity was observed. The meta-analysis results showed that rs1800629 was associated with RA in both Asian (*OR* = 2.17, 95% CI: 1.61–2.92, *P<*1.00E−4) and American (*OR* = 1.91, 95% CI: 1.40–2.62, *P<*1.00E−4) subgroups. However, there was different risk allele in the two subgroups (G allele for Asians and A allele for Americans). No evidence of association was observed in the European subgroup. This also indicated that regional difference is an important reason for the heterogeneity of rs7528684. The risk allele in Asian is G, while in American is A. For rs1800896, only the European subgroup was analyzed (the numbers of studies in the other subgroups were too small). There was a high heterogeneity in the European subgroup (*P* = 0.001) and rs1800896 is not associated with RA in the European subgroup. Further studies are required to identify the reasons behind the heterogeneity of the SNP rs1800896.

## Discussion

In the past two decades, many SNP loci have been identified as associated with RA by candidate gene association studies and GWAs. However, RA is a complex disease, and many genetic loci contribute to susceptibility to RA. Some association studies are underpowered for detecting the modest contributions of these genetic loci. This will lead to inconsistent results because of false-positives, false-negatives, or population differences [26], [27]. Meta-analysis is a powerful tool that can increase statistical power by pooling the results of independent studies [28] and, therefore, can improve the performance of genetic studies on complex diseases such as RA.

In this study, a comprehensive and systematic meta-analysis was carried out to assess the associations between 125 SNPs and RA susceptibility. Three genetic models were considered: the allele, recessive and dominant models. The meta-analysis results showed that 30, 28 and 26 SNPs were significantly associated with RA under each model, respectively. SNP rs2476601 had the strongest association with RA under all three models (*OR* = 1.605 for allele model, *OR* = 1.638 for dominant model, and *OR* = 2.544 for recessive model). The SNP is a common SNP, and is located in the *PTPN22* gene, which encodes a lymphoid-specific phosphatase (Lyp). rs2476601 is a nonsynonymous SNP, and changes the amino acid at position 620 from an arginine (R) to a tryptophan (W). This change affects the physical association with tyrosine kinase Csk during T cell activation [29], [30]. In addition to rs2476601, multiple SNPs in the *PTPN22* gene showed significant association with RA: five SNPs in the allele model (see Table 1); three in the dominant model (see Table 2); and four in the recessive model (see Table 3). These data are evidence of the association of *PTPN22* with RA. In addition, the *STAT4* and *PADI4* genes also had multiple SNPs associated with RA (STAT4: four, four and four SNPs for the allele, dominant and recessive models, respectively; PADI4: six, four and four SNPs for the allele, dominant and recessive models, respectively).

The publication bias for all the 125 SNPs was evaluated using funnel plots and Egger’s test. Only four SNPs showed significant bias: rs4810485 (allele, dominant, recessive model, P<1.00E−15, P<1.00E−15, P<1.00E−15), rs2280714 (dominant model, p = 0.006), TAP2 379A/G (allele model, p = 0.0003), and TNFRII 676T/G (allele model, p = 0.001). For all four sites, the results of the meta-analysis were not significant under all three genetic models. In other words, the significant association results in the meta-analysis were not affected by publication bias. This indicated that our meta-analysis is reliable. Nevertheless, some of the SNPs were tested for association only in a few studies, and there may be a publication bias for such SNPs. More studies on these loci are required.

In summary, a meta-analysis of 125 SNPs was carried out with the aim improving the statistical performance by increasing the sample size. After the meta-analysis, associations between certain SNPs and RA susceptibility were confirmed. However, certain SNP loci were reported by only a few articles, and further more studies are needed to clarify the associations between these SNPs and RA susceptibility.

## References

[1] Klareskog L, Catrina AI, Paget S (2009) Rheumatoid arthritis. Lancet 373: 659–672. 19157532 10.1016/S0140-6736(09)60008-8

[2] Kvien TK, Glennas A, Knudsrod OG, Smedstad LM, Mowinckel P, et al (1997) The prevalence and severity of rheumatoid arthritis in Oslo. Results from a county register and a population survey. Scand J Rheumatol 26: 412–418. 9433400 10.3109/03009749709065712

[3] Klareskog L, Padyukov L, Lorentzen J, Alfredsson L (2006) Mechanisms of disease: Genetic susceptibility and environmental triggers in the development of rheumatoid arthritis. Nat Clin Pract Rheumatol 2: 425–433. 16932734 10.1038/ncprheum0249

[4] MacGregor AJ, Snieder H, Rigby AS, Koskenvuo M, Kaprio J, et al (2000) Characterizing the quantitative genetic contribution to rheumatoid arthritis using data from twins. Arthritis Rheum 43: 30–37. 10643697 10.1002/1529-0131(200001)43:1<30::AID-ANR5>3.0.CO;2-B

[5] van der Woude D, Houwing-Duistermaat JJ, Toes RE, Huizinga TW, Thomson W, et al (2009) Quantitative heritability of anti-citrullinated protein antibody-positive and anti-citrullinated protein antibody-negative rheumatoid arthritis. Arthritis Rheum 60: 916–923. 19333951 10.1002/art.24385

[6] Hasstedt SJ, Clegg DO, Ingles L, Ward RH (1994) HLA-linked rheumatoid arthritis. Am J Hum Genet 55: 738–746. 7942852 PMC1918311

[7] Gregersen PK, Silver J, Winchester RJ (1987) The shared epitope hypothesis. An approach to understanding the molecular genetics of susceptibility to rheumatoid arthritis. Arthritis Rheum 30: 1205–1213. 2446635 10.1002/art.1780301102

[8] Raychaudhuri S (2010) Recent advances in the genetics of rheumatoid arthritis. Curr Opin Rheumatol 22: 109–118. 20075733 10.1097/BOR.0b013e328336474d PMC3121048

[9] Bowes J, Barton A (2008) Recent advances in the genetics of RA susceptibility. Rheumatology (Oxford) 47: 399–402. 18263596 10.1093/rheumatology/ken005

[10] Li Y, Begovich AB (2009) Unraveling the genetics of complex diseases: susceptibility genes for rheumatoid arthritis and psoriasis. Semin Immunol 21: 318–327. 19446472 10.1016/j.smim.2009.04.002

[11] Munoz-Valle JF, Valle Y, Padilla-Gutierrez JR, Parra-Rojas I, Rangel-Villalobos H, et al (2010) The +49A>G CTLA-4 polymorphism is associated with rheumatoid arthritis in Mexican population. Clin Chim Acta 411: 725–728. 20138855 10.1016/j.cca.2010.02.001

[12] Milicic A, Brown MA, Wordsworth BP (2001) Polymorphism in codon 17 of the CTLA-4 gene (+49 A/G) is not associated with susceptibility to rheumatoid arthritis in British Caucasians. Tissue Antigens 58: 50–54. 11580858 10.1034/j.1399-0039.2001.580110.x

[13] Lee YH, Rho YH, Choi SJ, Ji JD, Song GG (2007) PADI4 polymorphisms and rheumatoid arthritis susceptibility: a meta-analysis. Rheumatol Int 27: 827–833. 17265154 10.1007/s00296-007-0320-y

[14] Lee YH, Woo JH, Choi SJ, Ji JD, Song GG (2010) Association between the rs7574865 polymorphism of STAT4 and rheumatoid arthritis: a meta-analysis. Rheumatol Int 30: 661–666. 19588142 10.1007/s00296-009-1051-z

[15] Ji JD, Lee WJ, Kong KA, Woo JH, Choi SJ, et al (2010) Association of STAT4 polymorphism with rheumatoid arthritis and systemic lupus erythematosus: a meta-analysis. Mol Biol Rep 37: 141–147. 19479340 10.1007/s11033-009-9553-z

[16] Iwamoto T, Ikari K, Nakamura T, Kuwahara M, Toyama Y, et al (2006) Association between PADI4 and rheumatoid arthritis: a meta-analysis. Rheumatology (Oxford) 45: 804–807. 16449362 10.1093/rheumatology/kel023

[17] Totaro MC, Tolusso B, Napolioni V, Faustini F, Canestri S, et al (2011) PTPN22 1858C>T polymorphism distribution in Europe and association with rheumatoid arthritis: case-control study and meta-analysis. PLoS One 6: e24292. 21949702 10.1371/journal.pone.0024292 PMC3174938

[18] Ke-Wei R, Yuan-Yuan M, Nan-Wei X (2012) Reply: Clarification of Data for a Recent Meta-analysis: Meta-analysis of PTPN22 1858C/T Polymorphism and Rheumatoid Arthritis Risk in Europeans. Arch Med Res.

[19] Huedo-Medina TB, Sanchez-Meca J, Marin-Martinez F, Botella J (2006) Assessing heterogeneity in meta-analysis: Q statistic or I2 index? Psychol Methods 11: 193–206. 16784338 10.1037/1082-989X.11.2.193

[20] Ioannidis JP, Patsopoulos NA, Evangelou E (2007) Heterogeneity in meta-analyses of genome-wide association investigations. PLoS One 2: e841. 17786212 10.1371/journal.pone.0000841 PMC1950790

[21] Zintzaras E (2006) Methylenetetrahydrofolate reductase gene and susceptibility to breast cancer: a meta-analysis. Clin Genet 69: 327–336. 16630166 10.1111/j.1399-0004.2006.00605.x

[22] Higgins JP, Thompson SG, Deeks JJ, Altman DG (2003) Measuring inconsistency in meta-analyses. BMJ 327: 557–560. 12958120 10.1136/bmj.327.7414.557 PMC192859

[23] Lau J, Ioannidis JP, Schmid CH (1997) Quantitative synthesis in systematic reviews. Ann Intern Med 127: 820–826. 9382404 10.7326/0003-4819-127-9-199711010-00008

[24] Begg CB, Mazumdar M (1994) Operating characteristics of a rank correlation test for publication bias. Biometrics 50: 1088–1101. 7786990

[25] Egger M, Davey Smith G, Schneider M, Minder C (1997) Bias in meta-analysis detected by a simple, graphical test. BMJ 315: 629–634. 9310563 10.1136/bmj.315.7109.629 PMC2127453

[26] Lohmueller KE, Pearce CL, Pike M, Lander ES, Hirschhorn JN (2003) Meta-analysis of genetic association studies supports a contribution of common variants to susceptibility to common disease. Nat Genet 33: 177–182. 12524541 10.1038/ng1071

[27] Lee YH, Bae SC, Choi SJ, Ji JD, Song GG (2011) Associations between vitamin D receptor polymorphisms and susceptibility to rheumatoid arthritis and systemic lupus erythematosus: a meta-analysis. Mol Biol Rep 38: 3643–3651. 21110115 10.1007/s11033-010-0477-4

[28] Egger M, Smith GD (1997) Meta-Analysis. Potentials and promise. BMJ 315: 1371–1374. 9432250 10.1136/bmj.315.7119.1371 PMC2127866

[29] Vang T, Congia M, Macis MD, Musumeci L, Orru V, et al (2005) Autoimmune-associated lymphoid tyrosine phosphatase is a gain-of-function variant. Nat Genet 37: 1317–1319. 16273109 10.1038/ng1673

[30] Fiorillo E, Orru V, Stanford SM, Liu Y, Salek M, et al (2010) Autoimmune-associated PTPN22 R620W variation reduces phosphorylation of lymphoid phosphatase on an inhibitory tyrosine residue. J Biol Chem 285: 26506–26518. 20538612 10.1074/jbc.M110.111104 PMC2924087

